# Rapid clearance of *Borrelia burgdorferi* from the blood circulation

**DOI:** 10.1186/s13071-020-04060-y

**Published:** 2020-04-21

**Authors:** Liucun Liang, Jinyong Wang, Lucas Schorter, Thu Phong Nguyen Trong, Shari Fell, Sebastian Ulrich, Reinhard K. Straubinger

**Affiliations:** 1 Bacteriology and Mycology, Institute for Infectious Diseases and Zoonoses, Department of Veterinary Science, Faculty of Veterinary Medicine, LMU Munich, Munich, Germany; 2 Department of Microbiology & Immunology, Chicago Medical School, Rosalind Franklin University of Medicine and Science, North Chicago, USA; 3 Present Address: Shenzhen International Institute for Biomedical Research, Shenzhen, Guangdong People’s Republic of China; 4 Present Address: Chemisches Veterinäruntersuchungsamt Sigmaringen, Fidelis-Graf-Straße 1, 72488 Sigmaringen, Germany

**Keywords:** Lyme borreliosis, *Borrelia burgdorferi*, Tick-borne relapsing fever, *Borrelia persica*, Blood clearance

## Abstract

**Background:**

*Borrelia burgdorferi* is a tick-borne spirochete that causes Lyme borreliosis (LB). After an initial tick bite, it spreads from the deposition site in the dermis to distant tissues of the host. It is generally believed that this spirochete disseminates *via* the hematogenous route. *Borrelia persica* causes relapsing fever and is able to replicate in the blood stream. Currently the exact dissemination pathway of LB pathogens in the host is not known and controversially discussed.

**Methods:**

In this study, we established a strict intravenous infection murine model using host-adapted spirochetes. Survival capacity and infectivity of host-adapted *B. burgdorferi* sensu stricto (*Bbss*) were compared to those of *B. persica* (*Bp*) after either intradermal (ID) injection into the dorsal skin of immunocompetent mice or strict intravenous (IV) inoculation *via* the jugular vein. By *in vitro* culture and PCR, viable spirochetes and their DNA load in peripheral blood were periodically monitored during a 49/50-day course post-injection, as well as in various tissue samples collected at day 49/50. Specific antibodies in individual plasma/serum samples were detected with serological methods.

**Results:**

Regardless of ID or IV injection, DNA of *Bp* was present in blood samples up to day 24 post-challenge, while no *Bbss* was detectable in the blood circulation during the complete observation period. In contrast to the brain tropism of *Bp*, *Bbss* spirochetes were found in ear, skin, joint, bladder, and heart tissue samples of only ID-inoculated mice. All tested tissues collected from IV-challenged mice were negative for traces of *Bbss*. ELISA testing of serum samples showed that *Bp* induced gradually increasing antibody levels after ID or IV inoculation, while *Bbss* did so only after ID injection but not after IV inoculation.

**Conclusions:**

This study allows us to draw the following conclusions: (i) *Bp* survives in the blood and disseminates to the host’s brain *via* the hematogenous route; and (ii) *Bbss*, in contrast, is cleared rapidly from the blood stream and is a tissue-bound spirochete.
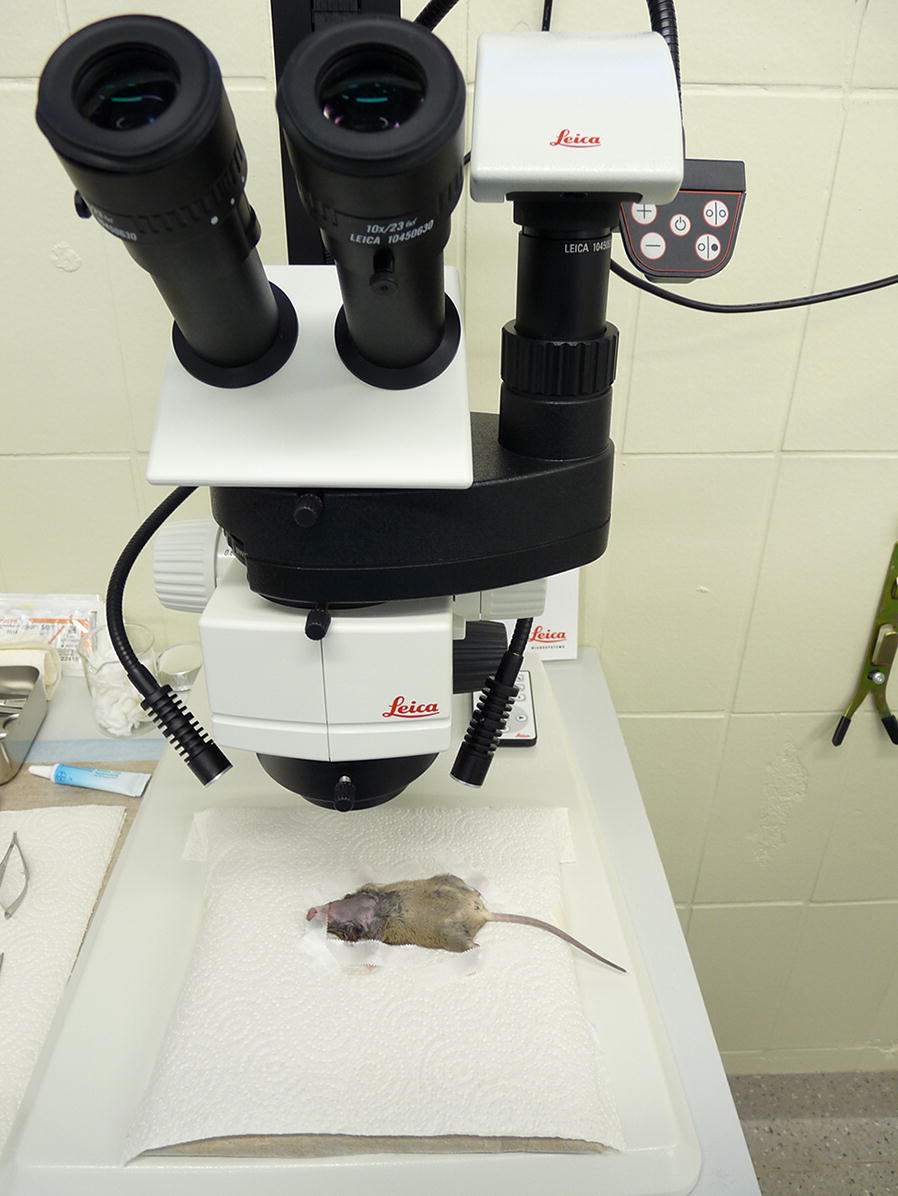

## Background

All known species of spiral-shaped bacteria (spirochetes) in the genus *Borrelia* are transmitted by ticks, except for *B. recurrentis*, which is transmitted by the human louse and leads to louse-borne relapsing fever (LBRF) [1]. Two groups of *Borrelia* stand out among these tick-borne species due to their prevalence as human pathogens [2, 3]. One group is spirochetes that are transmitted by fast-feeding argasid (soft) ticks of the genus *Ornithodoros* and cause tick-borne relapsing fever (TBRF). Among them, *B. persica* (*Bp*) is an important and prevalent pathogen of TBRF in humans [4] and is the main pathogen responsible for this disease in Central Asia and Middle East countries [5]. Clinically, recurrent episodes of high fever with (massive numbers of) spirochetes in the patient’s blood are a unique feature of the disease, whereas spirochetemia is not detected during afebrile periods [6, 7]. In addition, *Bp* causes infections in domestic dogs, cats [8] and, under experimental conditions, guinea pigs [9]. Assous et al. [10] detected borrelial organisms in blood specimens from mice four and six days after intraperitoneal (ip; intraabdominal) injection of blood samples from patients who had been diagnosed with having contracted a *Bp* infection. Furthermore, Addamiano & Babudieri [11] and Schwarzer et al. [12] discovered that *Bp* organisms reside in the brain tissue of infected mice late during the infection, while spirochetes were simultaneously not detectable in blood samples collected from the same animals. Despite this pathogenesis phenotype, little is known about the exact mechanisms how *Bp* crosses the endothelium barrier from the blood vessel into the host’s tissues. Similarly, the factors that are necessary to populate certain tissues types such as the brain are not known.

The other large group, Lyme borreliosis (LB) spirochetes, is transmitted by the slow-feeding ixodid (hard) ticks [2, 13–15]. Within the *B. burgdorferi* (*sensu lato*) complex, four genospecies have been identified as important human pathogens of LB. *Borrelia burgdorferi* sensu stricto (*Bb*ss) is found predominantly in the USA and less often in Europe, while *B. garinii*, *B. afzelii* and *B. bavariensis* occupy extensive regions in Eurasia [16, 17]. After ixodid ticks have deposited the *Borrelia* organisms in the skin, increasing spirochete numbers are found around the tick bite site, and they may initiate an early inflammatory reaction that is clinically evident as a rash (erythema migrans, EM). During later stages of infection, *B. burgdorferi* organisms spread to distant locations, resulting in a multisystem infectious disease (e.g. carditis and chronic arthritis) [18–20]. Clinical manifestations are thought to show following *Borrelia* dissemination [21–23]. In this context, some authors [24, 25] hold the view that *Borrelia* spirochetes use the blood stream, in which *B. burgdorferi* organisms first enter the vasculature near the deposition site after the tick bite and subsequently exit the vasculature to various tissues. Positive spirochete cultures and/or DNA detection of *B. burgdorferi* in plasma or blood samples from LB patients during the early stage of illness [21, 22, 26–28] are used as arguments to support the hypothesis of hematogenous dissemination of the organism. Other studies suggest, however, that dissemination of *B. burgdorferi* occurs by tissue migration rather than by blood stream dissemination since live spirochetes have been found with the highest frequency in tissues closest to the site of tick exposure [29]. Similarly, other investigators reported varying numbers of spirochetes and distinguished several degrees of joint and cardiac inflammation, which were strongly related to the inoculation site, e.g. the shoulder region *versus* footpad in experimental mice [30]. If the LB borreliae spread to further tissues through the blood stream, a random distribution of infection would be anticipated. Consequently, any joint might show signs of inflammation after it has been invaded by spirochete organisms. However, studies show that tissues closest to the tick bite show a higher probability to become infected and inflamed. For example, Berglund et al. [31] observed that “bites in the head and neck region were more common among children than among adults and were associated with an increased risk of neuroborreliosis”. Given this contradictory scientific background, it is necessary to determine whether LB pathogens disseminate *via* the blood stream, tissue migration, or both.

Nevertheless, Lyme borreliae may appear and can be found in the blood stream. Immunodeficient animals that lack mature T and B cells and complement [32] are known to show a high burden of *Bb*ss in their circulation due to their insufficient defense system [33–35]. The presence of spirochetes in the blood proves that the organisms may enter the circulation but is not an argument that spirochetes can actively leave the blood circulation and thus colonize distant sites of the body.

In the present study, we developed model by producing host-adapted *Bp* and *Bb*ss in immunodeficient mice and investigated their viability and infectivity in the blood stream of immunocompetent mice after intradermal (ID) and strict intravenous (IV) inoculation. The study aimed to clarify the dissemination pathways. Furthermore, whether LB *Borrelia* can spread *via* the bloodstream to distant tissue sites and establish an infection exclusively by the haematogenic route.

## Methods

### Mice

Specific pathogen-free, 6- to 8-week-old female NOD-SCID (non-obese diabetic-severe combined immune deficiency) and 8- to 10-week-old female C3H/HeOuJ (immunocompetent) mice were purchased from Janvier Labs (Saint Berthevin Cedex, France) and Charles River (Sulzfeld, Germany), respectively. All mice used herein were maintained and handled at the animal facility of the Institute of Infectious Diseases and Zoonoses, Ludwig-Maximilians-Universität (Munich, Germany). Mice were introduced into the animal facility at least one week prior to experiments to ensure adequate acclimation to the new environment. Two to three days prior to spirochete exposure, blood was collected from these NOD-SCID and C3H/HeOuJ mice, and plasma samples were used as baseline controls. After the experiment, all mice were sacrificed by cervical dislocation under anesthesia with medetomidine (0.5 mg/kg) combined with midazolam (5 mg/kg) [36, 37].

### Borrelial strains and cultivation conditions

Low-passage strains of *Bp* (LMU-C01; feline isolated, passage 2) and *Bb*ss (N40; passage 4) were used for the infection of mice [12, 38, 39]. Frozen glycerol stocks of *Bp* were cultivated in Pettenkofer/LMU *Bp* medium at 37 °C [40], and those of *Bb*ss were cultivated in commercial Barbour-Stoenner-Kelly H (BSK-H) complete medium with 6% rabbit serum (Sigma-Aldrich, Taufkirchen, Germany) at 33 °C [41]. Bacteria were counted with a dark-field microscope (10×/40; Leica DM2500; Leica Microsystems GmbH, Wetzlar, Germany) using a Petroff-Hausser counting chamber (Hausser Scientific, Horsham, Pennsylvania, USA). *Bp* and *Bb*ss were grown for 6 to 7 days to reach concentrations of 2.0 × 10^6^ and 1.0 × 10^7^ viable spirochetes per ml of medium, respectively. Total inocula of 1.0 × 10^5^ viable/motile *Bp* or 1.0 × 10^6^ viable/motile *Bb*ss organisms were prepared by adjusting the medium volume to 50 µl or 100 µl, respectively. All media used in this study contained no antibiotics, except for the media used for isolation of borreliae from blood samples, which contained 8 µg/ml kanamycin and 50 µg/ml rifampicin (Sigma-Aldrich), as described [42].

### Generation of host-adapted *Borrelia* organisms using immunodeficient mice

**Fig. 1:**
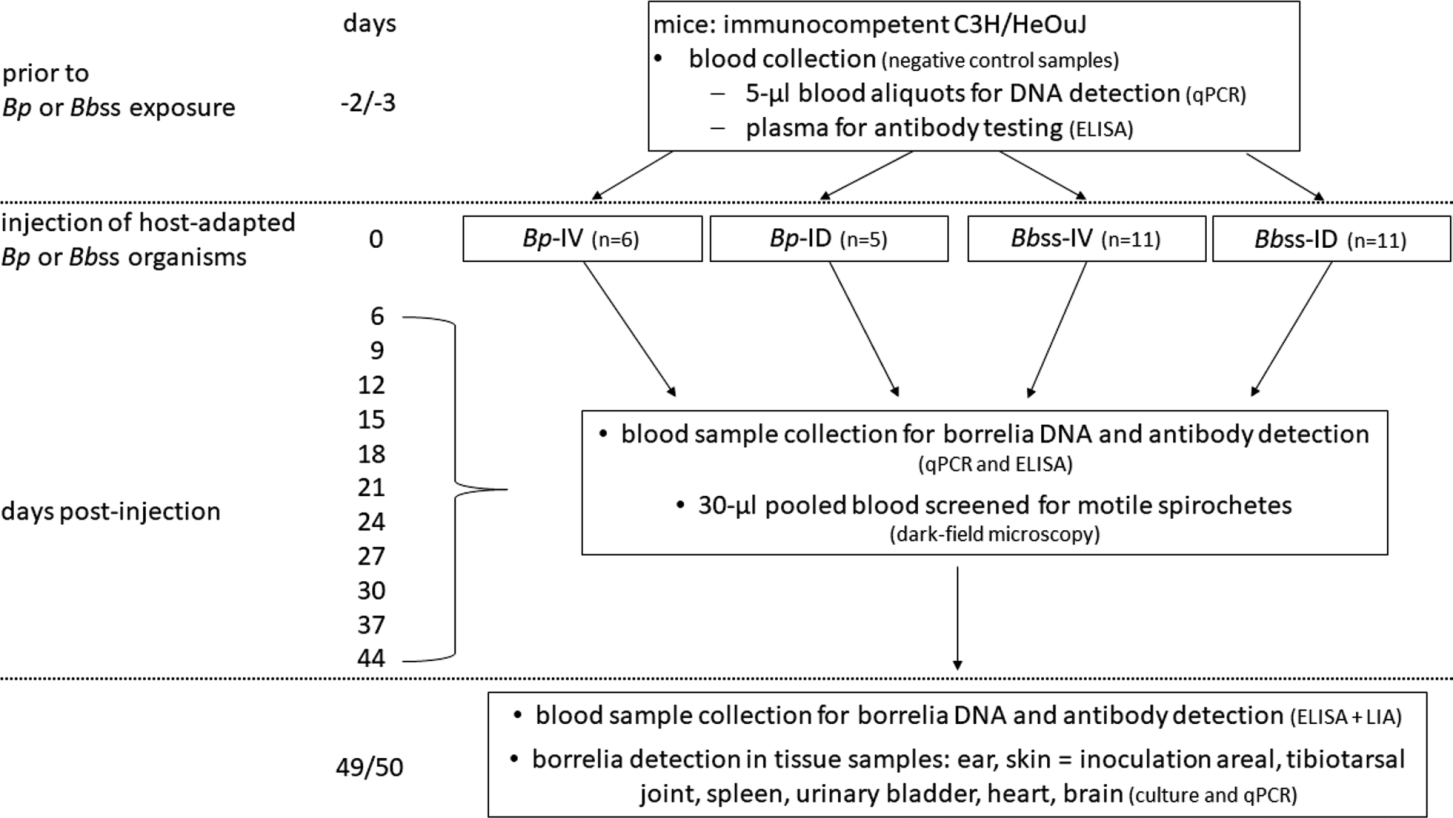
Timing of sampling and tests which were conducted with immunocompetent mice during the present study

Immunodeficient NOD-SCID mice were utilized to generate host-adapted *Borrelia* organisms due to their lack of mature T and B cells and complement. Compared to normal SCID mice, NOD-SCID mice have reduced macrophage and natural killer (NK) cell functions [32]. A flowchart with the timing of each sample and test is shown in Fig. 1. In the first step, six NOD-SCID mice each received intradermally 1.0 × 10^5^*Bp* or 1.0 × 10^6^*Bb*ss organisms (as described above) in their shaven dorsal backs (disinfected with 70% ethanol). Thereafter, these six mice were randomly divided into two equal subgroups, and blood samples were collected *via* facial vein puncture every other day for each subgroup until day 17. To confirm successful infection, 30 µl blood samples were diluted and mixed with 500 µl of medium for examination of motile spirochetes using dark-field microscopy, followed by cultivation of *Bp* or *Bb*ss in 5 ml of medium. In addition, the kinetics of the hematogenous spirochete load in the six NOD-SCID mice was documented with a qPCR test to determine the day of the largest bacterial load. A second separate group of animals consisting of five NOD-SCID mice was inoculated with an identical dose of spirochetes (*Bp* or *Bb*ss), as described above. On the anticipated day with the highest bacterial load, anticoagulated peripheral blood was collected aseptically with S-Monovette 2.7-ml K3E (Sarstedt AG & Co., Nümbrecht, Germany) by cardiac puncture under anesthesia. All blood samples from these five mice injected with *Bp* or *Bb*ss were pooled and subsequently checked for bacterial viability using a dark-field microscope. Aliquots from pooled blood samples were stored at − 30 °C for later qPCR analysis.

Additional tissue samples collected from ears, skin (inoculation areal), tibiotarsal joints, spleens, urinary bladders, hearts, and brains were aseptically removed on the anticipated day. Samples were divided into equal parts. Half of each tissue sample was transferred to medium for bacterial culture, and the other half was stored at − 30 °C for qPCR testing.

### Intradermal or strict intravenous injection of host-adapted *Borrelia* organisms into immunocompetent mice

Five C3H/HeOuJ mice served as negative control animals. These animals were anesthetized and received 100 µl of anticoagulated blood without *Borrelia* organisms collected from two non-infected NOD-SCID mice *via* strict IV injection into the jugular vein. Mice were monitored for five weeks; blood collection for plasma samples was performed once per week.

As scheduled, blood samples from individual immunodeficient NOD-SCID mice were collected and pooled together in the early morning, followed by microscopic inspection and aliquoting. Between approximately 10:00 h and 17:00 h, the pooled blood containing host-adapted spirochetes was then inoculated intravenously. Thereafter, intradermal injection was conducted in the evening. After the injection, the remaining pooled blood samples were inspected under dark-field microscopy and cultured in medium, and we confirmed the presence of motile spirochetes and their growth. Groups of C3H/HeOuJ mice received 100 µl of freshly pooled blood from five NOD-SCID mice, which contained host-adapted *Borrelia* spp. (*Bp* or *Bb*ss), as described above. These 100 µl aliquots from the same blood pool were injected into the shaven dorsal back (ID, disinfected with 70% ethanol) or jugular vein (IV) of C3H/HeOuJ mice. Injection experiments with host-adapted *Bb*ss organisms were repeated later with additional groups of C3H/HeOuJ mice (host-adapted organisms were obtained from another five NOD-SCID mice). All utilized C3H/HeOuJ mice (*n* = 33) were grouped as follows: 5 mice in the *Bp*-ID group and 6 mice in the *Bp*-IV group; 11 mice were allocated to *Bb*ss-ID (5 in the first group; 6 in a second group); and 11 mice were allocated to *Bb*ss-IV (5 in the first group; 6 in a second group).

The surgery for intravenous spirochete injection was carried out as follows. All mice were deeply anesthetized by IP injection of medetomidine (0.5 mg/kg), midazolam (5 mg/kg) and fentanyl (0.05 mg/kg) [36, 37]. The anesthesia protocol was the same for all mice. A warming pad (model #39DP; Braintree Scientific, Inc., Braintree, USA) was used to maintain the body temperature at 37 °C. Hair under the chin was removed by shaving, and this area was disinfected with 70% ethanol and air-dried. At a position slightly right of the body’s midline under the chin, an ~ 1 cm long incision was made into the skin, and the tissue was carefully dissected to obtain access to the right jugular vein. Using a stereomicroscope (Leica M60; Leica Microsystems GmbH) equipped with a Leica MC170 HD camera (Leica Microsystems GmbH) and two gooseneck lights (Leica LED3000 SLI; Leica Microsystems GmbH), connective tissue and fat were further removed without injuring the jugular vein. Splinter forceps (No. 310645; Henry Schein Vet GmbH, Hamburg, Germany) and eye forceps (No. 310174; Henry Schein Vet GmbH) were utilized during this procedure. The exposed vein and removed tissue were immersed in sterile physiological saline. By holding the jugular vein with a bulb-headed probe (No. 310335; Henry Schein Vet GmbH), two loop ligations were made around the vein with Surgicryl® PGA polyglycolic acid suture (SMI AG, St. Vith, Belgium). The up-ligation was completely closed, and the down-ligation was a loose knot fixed with Student Halsted-Mosquito Hemostats (No. 91309-12; Fine Science Tools GmbH, Heidelberg, Germany). An injection catheter was fully filled with sterile 0.9% saline (~ 13 µl). The jugular vein was positioned with two bulb-headed probes to avoid damage and bleeding. Vannas-style spring scissors (No. 15000-03; Fine Science Tools GmbH) were used to cut a 45° angle hole (up to down) into the jugular vein. A micro hook (blunt, No. 10062-12; Fine Science Tools GmbH) was a superb tool for opening and grabbing the vessel’s wall and for inserting the catheter. Intravenous injection was performed with an Alzet Mouse Jugular Catheter (No. 0007700; Durect Corporation, California, USA) with a 23-gauge Terumo Agani needle (Shanghai International Holding Corp. GmbH, Hamburg, Germany) attached to a 1 ml single-use syringe (Dispomed Witt oHG, Gelnhausen, Germany) filled with 140 µl of pooled blood from the immunodeficient donor mice. When ~ 9 mm of the catheter tip was introduced into the jugular vein, the down-ligation was closed and secured such that the catheter would not slip out from the vein. The down-placed bulb-headed probe was removed for adequate space and fluent injection. After attaching the 23-gauge needle (connected with the 1 ml syringe) to the exposed end of the catheter, 100 µl of blood was injected very slowly (~ 10 µl/min) into the vein. Thereafter, the needle was removed from the catheter, and a second 23-gauge needle connected with the 1 ml syringe filled with sterile 0.9% saline was subsequently attached to the catheter. The catheter was flushed with 50 µl of sterile saline. Then, the catheter tip was carefully pulled back and was still connected to the needle and syringe. At the same time, the down-ligation was entirely fastened, avoiding any blood loss into the tissue. The incision site was closed with an intracutaneous suture (4–5 single sutures) using 5/0 Monosyn® Easyslide (DS16; B. Braun Surgical SA, Barcelona, Spain). Atipamezole (2.5 mg/kg), flumazenil (0.5 mg/kg) and naloxone (1.2 mg/kg) [36, 37] were injected intraperitoneally. Standard aseptic techniques were employed during the complete procedure. Videos documenting the surgical progress and the strict IV injection of blood samples were recorded through 10×/1.6 objectives of the stereomicroscope.

### Blood, plasma and serum sample collection and cultivation of spirochetes

After ID or IV injection, blood samples were collected *via* facial vein puncture from the C3H/HeOuJ mice. Two to four drops of blood were transferred directly into a Microvette 100 K3E (preparation K_3_EDTA; Sarstedt AG & Co.). First blood samples were collected on day 6 post-infection (pi) (recovery time after surgery) and thereafter every three days until day 30 pi. After day 30 pi, additional blood samples were collected on days 37, 44, and 49/50 pi. Five microliters of blood from each mouse at each time point was stored for qPCR screening. Plasma samples were harvested from pooled blood in each IV or ID group by centrifugation at 350× *g* for 10 min at 23 °C. Serum samples were obtained from a large volume of blood sampled *via* cardiac puncture from each individual mouse under anesthesia on day 49/50 pi. Mice were collected because of technical reasons either on day 49 pi or on day 50 pi. All the samples were frozen at − 30 °C for subsequent tests.

Thirty microliters of pooled blood samples were used to monitor and cultivate spirochetes in 5 ml of medium. Cultures were inspected for the presence of motile spirochetes using a dark-field microscope (10×/40; Leica DM2500; Leica Microsystems GmbH).

### Tissue samples for spirochete cultivation and qPCR

On day 49/50 post-spirochete inoculation, tissue samples from the ears, skin (site of inoculation), tibiotarsal joints, spleens, urinary bladders, hearts, and brains were aseptically removed from each euthanatized C3H/HeOuJ mouse. Tissue samples were immersed in 70% ethanol for 1 min and then washed with sterile phosphate-buffered saline (PBS) for 30 s. Ear and skin tissues were immersed in 70% ethanol for 2 min. After washing, tissues were cut into two approximately equal parts. Half of each sample was placed in a 1.5 ml microcentrifuge tube and frozen at −30 °C for later DNA extraction. For cultivation of spirochetes, the other tissue portion was transferred into a sterile Stomacher closure bag (Seward Laboratory, London, UK) containing 2 ml of Pettenkofer/LMU *Bp* medium without antibiotics for *Bp* or 2 ml of BSK-H complete medium without antibiotics for *Bb*ss. Stomacher bags were processed at normal speed for 60 s with a Stomacher® 80 *micro*Biomaster (Seward Laboratory). Subsequently, the medium and tissue samples were transferred into a 12 ml screw top tube (centrifuge tube 12; TPP, Faust Lab Science GmbH, Klettgau, Germany) prefilled with 8 ml of the same medium. Cultures were incubated at 37 °C (*Bp*) or 33 °C (*Bb*ss) for six weeks and examined once per week with a dark-field microscope (10×/40; Leica DM2500; Leica Microsystems GmbH).

### Detection of borrelial DNA in murine blood and tissue samples

All blood and tissue samples from the NOD-SCID and C3H/HeOuJ mice were subjected to DNA extraction and PCR detection according to published protocols [12]. The detection limits were 6.9 spirochetes/µl blood (Cq value of 39.900) and 3.0 spirochetes/mg tissue (Cq value of 39.372). A QuantStudio 5 real-time qPCR system (Applied Biosystems, ThermoFisher Scientific GmbH, Ulm, Germany) was used to amplify the DNA target genes *flaB* (*Bp*) [12] and *ospA* (*Bb*ss) [43]. Oligonucleotide primer pairs and probes (Table 1) were synthesized by Eurofins Genomics (Ebersberg, Germany). Each qPCR reaction was set up in a 20 µl final volume (Table 2). The PCR programs consisted of (i) heating at 95 °C for 2 min for polymerase activation and DNA denaturation; (ii) amplification for 40 cycles with denaturation at 95 °C for 5 s and extension and annealing at 60 °C for 25 s; and (iii) a final step at 25 °C for 2 min in a 96 multiply PCR plate natural (Sarstedt AG & Co.).

**Table 1:** Primers and probes utilized in this study

Primer name	Sequence (5′-3′)
*Bp*_flaB_fw	GAGGGTGCTCAACAAGCAA
*Bp*_flaB_re	CAACAGCAGTTGTAACATTAACTGG
*Bp*_flaB_probe	FAM-AAATCAGGAAGGAGTACAACCAGCAGCA-TAM
*Bb*ssN40-ospA 17 fw	AATGTTAGCAGCCTTGACGAGAA
*Bb*ssN40-ospA 119 re	GATCGTACTTGCCGTCTTTGTTT
*Bb*ssN40-ospA-41T	FAM-AACAGCGTTTCAGTAGATTTGCCTGGTGA-TAM

**Table 2:** PCR mixture used in this study

PCR	*Bp* (*flaB*)		*Bb*ss (*ospA*)	
	Working concentration	Reaction volume (µl)	Working concentration	Reaction volume (µl)
Master mix^a^	1×	10	1×	10
Forward primer	600 nM	1.2	900 nM	0.64
Reverse primer	600 nM	1.2	900 nM	0.64
Probe	200 nM	0.8	100 nM	0.97
Reference dye^b^		0.1	–	–
Nuclease-free water		4.2		5.25
Template DNA		2.5		2.5

^a^QuantiNova probe PCR master mix (Qiagen, Hilden, Germany)

^b^QN ROX reference dye (Qiagen)

*Abbreviations*: *Bb*ss, *Borrelia burgdorferi* sensu stricto; *Bp*, *Borrelia persica*

To assess the copy numbers of the target genes in the blood and tissue samples, PCR-based standard curves were established with known amounts of double-stranded DNA (dsDNA) of the *flaB* or *ospA* gene synthesized by Metabion International AG (Planegg, Germany). Tenfold serial dilutions were made ranging from 1.0 × 10^7^ to 10^1^ copies per reaction. Two positive controls of dsDNA (containing 1.0 × 10^3^ and 1.0 × 10^5^ copies of the *Bp* or *Bb*ss target gene) and a no template control (NTC, 2.5 µl of nuclease-free water) were included in each run. Both standard template dsDNA and sample DNA from mouse blood and tissues were amplified in triplicate. Based on the obtained quantification cycle (Cq), the number of spirochetes per ml of blood or per mg of tissue was calculated using QuantStudio Design and Analysis Software (Applied Biosystems).

### Antibody levels measured with a kinetic ELISA

ELISA plates were coated with whole-cell lysates from *Bp* or *Bb*ss cultures, which had been sonicated and prepared as previously described [44, 45]. Pooled plasma samples and serum samples from individual mice were diluted at 1:100 with sample buffer (PBS) containing 0.05% Tween 20 (AppliChem GmbH, Darmstadt, Germany) and 2% nonfat dry milk (Merck KGaA, Darmstadt, Germany). A computerized kinetic ELISA was applied as described previously [46]. Peroxidase-conjugated goat anti-mouse immunoglobulins (IgG, IgA, and IgM; MP Biomedicals, LLC, Heidelberg, Germany) at a dilution of 1:4000 (for *Bp*) and 1:3000 (for *Bb*ss) served as a secondary detection antibody. Each test included negative and positive controls. All plasma and serum samples were tested in duplicate, and mean values are reported.

### Visualization of specific antibodies against *Bb*ss *via* line immunoassay

Serum samples from individual mice collected on day 49/50 pi with *Bb*ss were analyzed with a line immunoblot assay (LIA) to visualize specific antibodies against this agent. Briefly, IgG immunoblotting strips (Sekisui Virotech GmbH, Rüsselsheim, Germany) with the recombinant protein fractions (VlsE mix, OspA mix, DbpA mix, OspC mix, BmpA, p58, and p83/100) were prepared according to the manufacturer’s instructions. Serum samples were diluted 1:100 in the ready-to-use IgG immunoblot dilution/wash buffer (Sekisui Virotech GmbH). The same secondary antibody used in the ELISA was applied at a dilution of 1:1000, and strips were incubated for 30 min at room temperature. After three washing steps with the dilution/wash buffer and one time with distilled water, the color reaction was achieved by adding substrate solution (Opti-4CN Substrate Kit; Bio-Rad Laboratories GmbH, Munich, Germany) and stopped by washing the strips with distilled water three times. Images were taken with a Cemi-DocMP System and Image Lab Software Version 5.0 (Bio-Rad Laboratories GmbH).

### Statistical analysis

All graphs in this study were prepared with OriginPro 2017 Software (Additive GmbH, Friedrichsdorf, Germany). Data are presented as the means and standard deviations (SDs).

## Results

### Population kinetics of host-adapted *Borrelia* in the blood of NOD-SCID mice

**Fig. 2:**
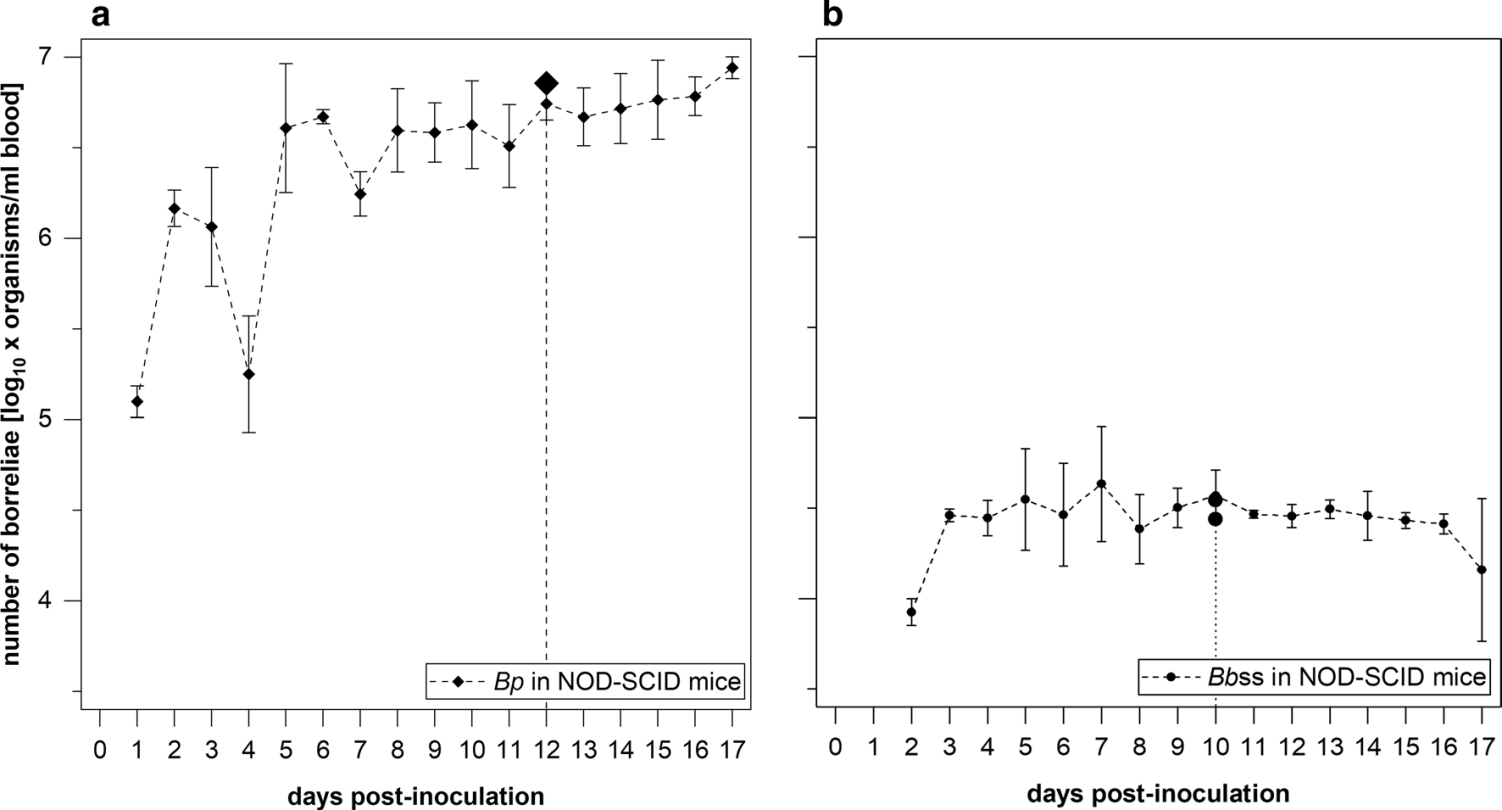
Kinetics of host-adapted *Borrelia* spp. in the blood of immunodeficient NOD-SCID mice over a course of 17 days. Mice were injected intradermally with cultured 1.0 × 10^5^*Bp* (**a**) or 1.0 × 10^6^*Bbss* (**b**) per animal. **a***Bp* was detectable on day 1 pi, with an increasing hematogenous spirochete burden over time. On day 12 pi, an average of 5.6 ± 1.2 × 10^6^ cells were detected per ml blood among six NOD-SCID mice. In five additional NOD-SCID mice, 7.2 × 10^6^*Bp*/ml blood was detected on day 12 pi. **b** The first *Bbss* in blood were detectable on day 2 (8.5 ± 1.4 × 10^3^ cells/ml), and concentrations climaxed at 4.9 ± 3.3 × 10^4^ cells/ml blood on day 7, plateauing at ~ 3.0 × 10^4^ until day 17 pi. On day 10 pi, the average borrelial load was 3.9 ± 1.3 × 10^4^ organisms per ml blood among six NOD-SCID mice. In two additional groups of five NOD-SCID mice, 3.5 × 10^4^ and 2.8 × 10^4^*Bbss*/ml blood were detected on day 10 pi

Two groups of six NOD-SCID mice each were challenged independently with an intradermal inoculation of *in vitro* cultured 1.0 × 10^5^*Bp* and 1.0 × 10^6^*Bb*ss. The bacterial burden and viability of host-adapted *Borrelia* organisms were evaluated. On day 1 pi, the *flaB* gene of *Bp* (1.3 ± 0.2 × 10^5^ copies/ml) was detectable in the blood samples from mice receiving *Bp*, and viable spirochetes were observed in blood cultures from all six mice (30 µl of blood in corresponding cultures). The number of *Bp* kept increasing over time, although some minor declines occurred on day 4 (2.2 ± 1.7 × 10^5^ cells/ml), day 7 (1.8 ± 0.5 × 10^6^ cells/ml), and day 11 (3.6 ± 2.0 × 10^6^ cells/ml; Fig. 2a). Considering the numbers and viability of *Bp* organisms observed at various time points; day 12 (5.6 ± 1.2 × 10^6^ cells/ml blood) was identified as the optimal sampling time point to obtain spirochete-containing blood samples to be used for the subsequent challenge of immunocompetent C3H/HeOuJ mice. An additional five NOD-SCID mice were inoculated with *Bp*. Blood with 7.2 × 10^6^*Bp* per ml was harvested on day 12 pi (Fig. 2a). In the other group, *Bb*ss organisms were detectable for the first time on day 2 (8.5 ± 1.4 × 10^3^ cells/ml) and reached 4.9 ± 3.3 × 10^4^ cells/ml blood on day 7, plateauing at a concentration of ~ 3.0 × 10^4^ cells/ml blood until day 17 pi (Fig. 2b). On day 10, sufficient numbers of spirochetes for subsequent infection experiments (3.9 ± 1.3 × 10^4^ cells/ml) were detected in blood samples. Two additional groups with five NOD-SCID mice each were inoculated intradermally with *Bb*ss. On day 10 pi, blood from the five mice in each group was pooled, resulting in 3.5 × 10^4^ and 2.8 × 10^4^*Bb*ss organisms per ml blood (Fig. 2b).

### Distribution of host-adapted borreliae in tissues of immunodeficient NOD-SCID mice

Tissue samples collected on day 12 pi (*Bp*) or day 10 pi (*Bb*ss) from the ear (only in the case *Bb*ss-inoculated mice), skin (site of inoculation), tibiotarsal joint, spleen, urinary bladder, heart, and brain of each NOD-SCID were positive for borrelial DNA. In preceding (data not shown) experiments, the growth kinetics of *Borrelia* cultures was evaluated. The days 12 and 10 produced *Borrelia* in sufficient numbers and quality in the case of *Bbss* and *Bp*, respectively. Motile spirochetes, *Bp* and *Bb*ss, were detected in culture medium cultivated with samples collected from the joints, spleens, bladders, hearts and brains. Detection of *Bp* and *Bb*ss by culture failed in the case of all the ear and skin samples due to extensive bacterial contamination and overgrowth (Table 3).

**Table 3:** Distribution of *Borrelia persica* and *Borrelia burgdorferi* sensu stricto spirochetes in tissues of immunodeficient NOD-SCID mice on day 49/50

Inoculum^a^	*n*	Spirochetes in tissue samples detected by culture/qPCR (organisms/mg)						
		Ear	Skin	Joint	Spleen	Bladder	Heart	Brain
Intradermal								
*B. persica* (1.0 × 10^5^)		#/0	#/17	#/199	+/873	+/74,518	+/14,017	+/3702
		#/0	#/47,690	#/117	#/19	#/1743	#/46,102	#/10,721
		#/0	#/11,529	+/262	−/3381	+/83,700	+/23,582	+/11,929
		#/0	#/1171	#/574	+/1050	+/26,254	#/28,797	+/2639
		#/0	#/311	#/24	#/144	#/131,872	#/6168	#/1896
Positive mice	3/5	0/0	0/5	1/5	2/5	3/5	2/5	3/5
Intradermal								
*B. burgdorferi* sensu stricto (1.0 × 10^6^)		#/48	#/41,467	+/20,395	+/22	+/266,594	+/360	+/11
		#/522	#/18,795	+/37,393	+/537	+/217,600	#/333	+/12
		#/3082	#/16,655	#/2113	#/14	+/2,110,835	+/357	+/15
		#/10,372	#/18,435	+/40,571	#/3	+/1,141,583	+/1122	+/47
		#/4,987	#/15,288	+/17,693	+/992	+/384,204	+/2057	+/12
Positive mice	5/5	0/5	0/5	4/5	3/5	5/5	4/5	5/5

^a^Culture-derived borreliae

*Key*: #, contaminated culture; +, positive, with motile spirochetes in culture; −, negative, no spirochetes in culture

### Spirochetemia in immunocompetent C3H/HeOuJ mice

**Fig. 3:**
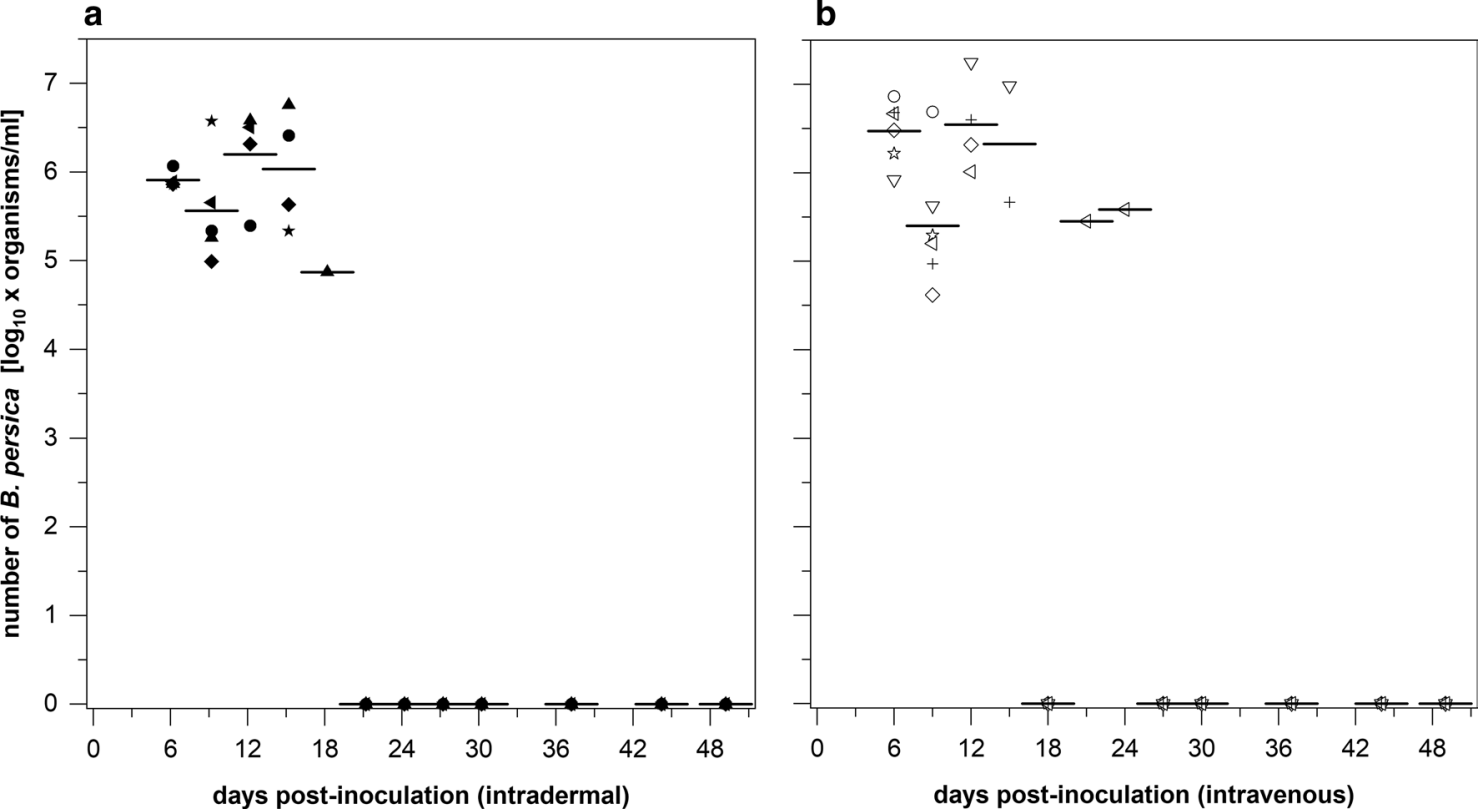
Kinetics of *Bp* DNA in the blood of immunocompetent C3H/HeOuJ mice during a course of 49/50 days. C3H/HeOuJ mice were challenged (**a**) intradermally or (**b**) *via* a strict intravenous route with 7.2 × 10^5^ host-adapted *Bp* spirochetes per animal. Blood samples were screened with a qPCR test method. Between day 27 and days 49/50, no spirochetal DNA was detected in blood samples of any mouse. Different symbols indicate levels in individual mice and horizontal lines indicate mean values per sampling day

One hundred microliters of pooled blood that contained 7.2 × 10^5^ host-adapted *Bp* or 3.5/2.8 × 10^3^ host-adapted *Bb*ss organisms was injected ID or strictly IV into C3H/HeOuJ mice. Throughout a 49/50-day study period, blood samples were collected at defined time points. All mice inoculated with *Bp* intradermally or intravenously showed *Borrelia* DNA signals until day 15 pi (Fig. 3); viable spirochetes were also observed in the blood during this period by dark-field microscopy. Bacterial concentrations increased in the blood to 8.3 ± 1.9 × 10^5^ (day 6) and 1.9 ± 1.7 × 10^6^ (day 12) organisms per ml in mice inoculated with *Bp* intradermally. Only one single animal inoculated intradermally with *Bp* was positive until day 18 (7.4 × 10^4^ cells/ml blood). Similarly, the mice inoculated intravenously with *Bp* demonstrated two peaks in spirochete population kinetics: 3.7 ± 2.4 × 10^6^ cells/ml blood on day 6 and 4.1 ± 6.8 × 10^6^ cells/ml blood on day 12. One single mouse produced 2.8 × 10^5^ and 3.8 × 10^5^*Bp* per ml blood on days 21 and 24, respectively (Fig. 3). Between day 27 and days 49/50, however, all mice were negative for *Borrelia* DNA, and no motile *Bp* organisms were recovered from 30 µl blood samples.

In contrast, *Bb*ss was not visible microscopically (dark-field) in blood samples collected from C3H/HeOuJ mice or in their pooled blood sample batches, regardless of whether the animals had been exposed to the spirochetes *via* the ID or IV route. Cultivation of 30 µl blood samples in 5 ml of BSK-H medium showed no growth of *Bb*ss spirochetes over the period of a six-week incubation. Additionally, not a single *Bb*ss-specific DNA signal was detected in any blood specimens collected from all immunocompetent C3H/HeOuJ mice after *Bb*ss inoculation via the ID or IV route. This finding was highly statistically significant (Fisher’s exact test, *P* < 0.00001).

### *Borrelia* distribution in tissues of immunocompetent C3H/HeOuJ mice

*Bp* organisms were observed in all cerebral tissue samples collected from immunocompetent C3H/HeOuJ mice (*n* = 11) on day 49/50 post-ID or IV spirochete inoculation (100%). The other tissue samples (those from the joint, spleen, urinary bladder, and heart) produced neither viable spirochetes in culture medium nor *flaB* gene signals in the PCR tests (Table 4). Bacterial contamination occurred in cultures with ten ear and eight skin tissue samples, while the rest of the samples (one ear and three skin tissue samples) showed no *Bp* growth (Table 4).

**Table 4:** Distribution of *Borrelia persica* spirochetes in tissues of immunocompetent C3H/HeOuJ mice on day 49/50

Inoculum^a^	*n*	Spirochetes in tissue samples by culture/qPCR (organisms/mg)						
		Ear	Skin	Joint	Spleen	Bladder	Heart	Brain
Intradermal								
*B. persica* (7.2 × 10^5^)		#/0	#/0	−/0	−/0	−/0	−/0	+/39
		#/0	#/0	−/0	−/0	−/0	−/0	+/31
		#/0	−/0	−/0	−/0	−/0	−/0	+/89
		#/0	#/0	−/0	−/0	−/0	−/0	+/59
		#/0	−/0	−/0	−/0	−/0	−/0	+/136
Positive mice	5/5	0/0	0/0	0/0	0/0	0/0	0/0	5/5
Intravenous								
*B. persica* (7.2 × 10^5^)		#/0	#/0	−/0	−/0	−/0	−/0	+/30
		#/0	#/0	−/0	−/0	−/0	−/0	+/34
		−/0	−/0	−/0	−/0	−/0	−/0	+/75
		#/0	#/0	−/0	−/0	−/0	−/0	+/216
		#/0	#/0	−/0	−/0	−/0	−/0	+/63
		#/0	#/0	−/0	−/0	−/0	−/0	+/74
Positive mice	6/6	0/0	0/0	0/0	0/0	0/0	0/0	6/6

^a^Host-adapted borreliae

*Key*: #, contaminated culture; +, positive, with motile spirochetes in culture; −, negative, no spirochetes in culture

When *Bb*ss was injected intradermally into immunocompetent C3H/HeOuJ mice, 100% of these animals were positive 50 days after the inoculation of host-adapted spirochetes, as shown by culture and qPCR (Table 5). Motile *Bb*ss organisms were observed in medium cultured with tissue specimens from ears (4/11), skin samples (10/11), joints (11/11), spleens (4/11), bladders (11/11), hearts (11/11) and brains (2/11). *Bb*ss-specific DNA was detected only in urinary bladders (11/11) and heart tissue samples (7/11) of these animals (Table 5). In strict contrast, all eleven mice that had intravenously received the host-adapted borreliae tested completely negative for viable *Bb*ss organisms and borrelial DNA in any tissue sample (Table 5).

**Table 5:** Distribution of *Borrelia burgdorferi* sensu stricto spirochetes in tissues of immunocompetent C3H/HeOuJ mice on day 49/50

Inoculum^a^	*n*	Spirochetes in tissue samples by culture/qPCR (organisms/mg)						
		Ear	Skin	Joint	Spleen	Bladder	Heart	Brain
Intradermal								
*B. burgdorferi* sensu stricto (3.5/2.8 × 10^3^)		#/0	+/0	+/0	#/0	+/875	+/0	−/0
		#/0	+/0	+/0	+/0	+/676	+/0	+/0
		+/0	+/0	+/0	+/0	+/1041	+/409	−/0
		#/0	+/0	+/0	+/0	+/1867	+/0	+/0
		#/0	#/0	+/0	+/0	+/1482	+/0	−/0
		+/0	+/0	+/0	−/0	+/2420	+/893	−/0
		+/0	+/0	+/0	−/0	+/10,295	+/501	−/0
		+/0	+/0	+/0	−/0	+/1383	+/788	−/0
		#/0	+/0	+/0	−/0	+/2741	+/817	−/0
		#/0	+/0	+/0	−/0	+/906	+/109	−/0
		#/0	+/0	+/0	−/0	+/3487	+/607	−/0
Positive mice	11/11	4/0	10/0	11/0	4/0	11/11	11/7	2/0
Intravenous								
*B. burgdorferi* sensu stricto (3.5/2.8 × 10^3^)		#/0	−/0	−/0	−/0	−/0	−/0	−/0
		#/0	−/0	−/0	−/0	−/0	−/0	−/0
		#/0	#/0	−/0	−/0	−/0	−/0	−/0
		#/0	−/0	−/0	−/0	−/0	−/0	−/0
		#/0	−/0	−/0	−/0	−/0	−/0	−/0
		−/0	#/0	−/0	−/0	−/0	−/0	−/0
		−/0	#/0	−/0	−/0	−/0	−/0	−/0
		−/0	#/0	−/0	−/0	−/0	−/0	−/0
		#/0	#/0	−/0	−/0	−/0	−/0	−/0
		#/0	#/0	−/0	−/0	−/0	−/0	−/0
		#/0	#/0	−/0	−/0	−/0	−/0	−/0
Positive mice	0/0	0/0	0/0	0/0	0/0	0/0	0/0	0/0

^a^Host-adapted borreliae

*Key*: #, contaminated culture; +, positive, with motile spirochetes in culture; −, negative, no spirochetes in culture

### Detection of specific antibodies against *Bp* and *Bb*ss

**Fig. 4:**
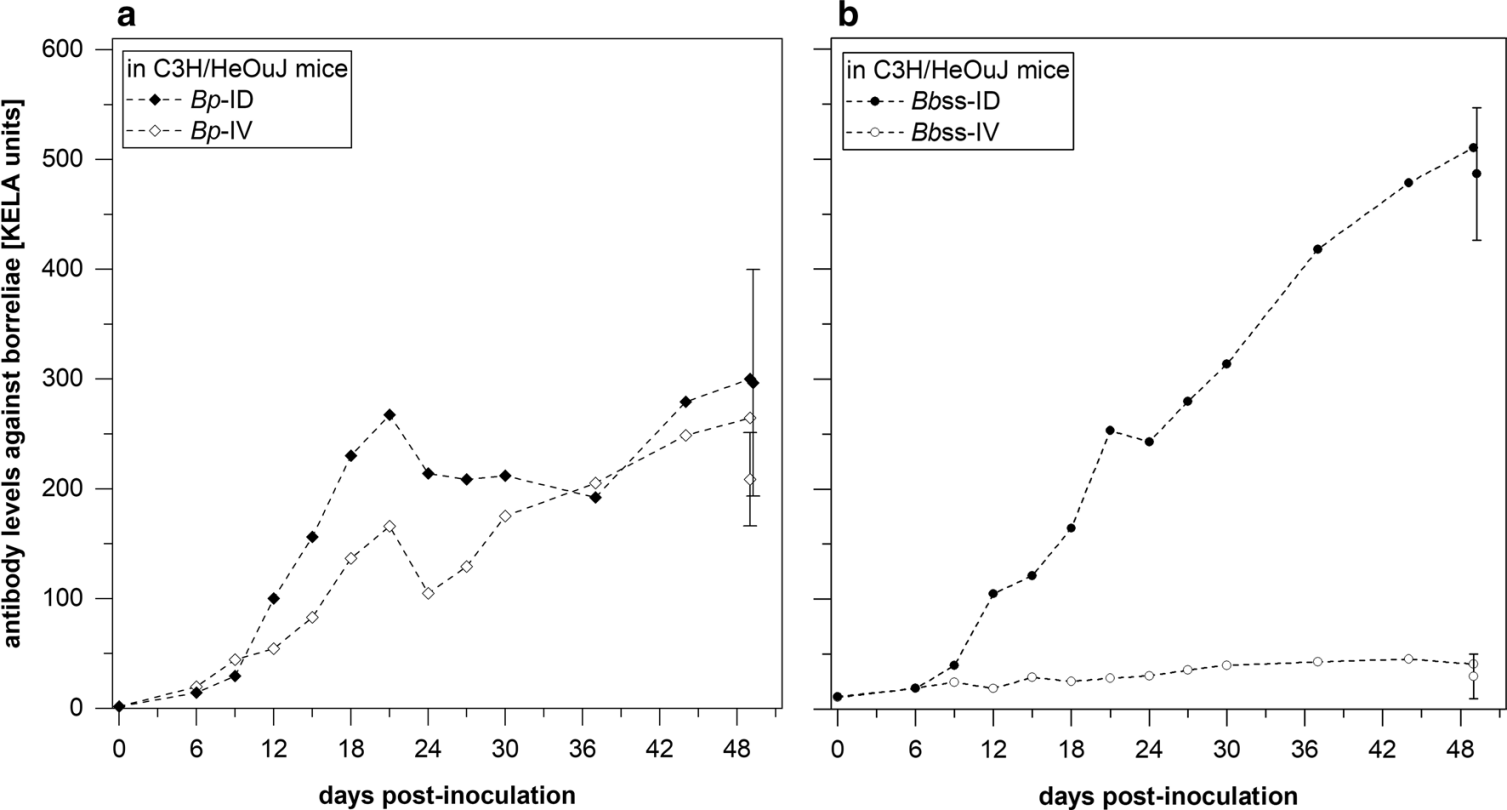
Specific antibodies against borrelia organisms in C3H/HeOuJ mice serum. Specific antibodies against *Bp* (**a**) or *Bbss* (**b**) were induced in C3H/HeOuJ mice. **a** Both groups of mice, inoculated intradermally or intravenously with *Bp*, showed rising antibody levels until day 21 pi, followed by a slight decrease and a repeated increase until day 49/50 pi. Data for individual serum samples from single animals are represented for days 49/50 pi (bar symbols). **b** Mice inoculated intradermally with *Bbss* showed increasing levels of specific antibodies against *Bbss*. Data for individual serum samples from single animals are represented for days 49/50 pi (bar symbols). In contrast, specific antibodies against *Bbss* were not detected when mice received *Bbssvia* strict intravenous injection

To monitor the adaptive immune response against borrelia organisms during the infection period, *Bp*- or *Bb*ss-specific antibody levels were measured with a kinetic ELISA (KELA). As shown in Fig. 4a, injection (ID and IV) of host-adapted *Bp* into immunocompetent C3H/HeOuJ mice elicited strong antibody responses. In both groups, antibody levels rose to 267.5 and 166.1 mean KELA (a computerized kinetic enzyme-linked immunosorbent assay) units on day 21 and decreased to 214.1 and 104.9 KELA units on day 24, respectively. Thereafter, antibody levels reached 300.1 (ID) and 264.5 (IV) KELA units, respectively until days 49/50. Due to the larger-volume serum samples obtained at necropsy on days 49/50, each animal was tested individually, and together, they produced mean values of 296.6 ± 103.1 (ID) and 208.7 ± 42.5 KELA units (IV).

Mice that had been injected intradermally with host-adapted *Bb*ss organisms (both those injected with 3.5 and 2.8 × 10^3^ organisms/animal) showed a steady increase in antibody levels up to 510.3 mean KELA units on day 49/50 pi. Serum samples from individual animals collected on the final day of the experiment responded clearly in the ELISA, with 486.4 ± 60.2 units (Fig. 4b). In contrast, none of the mice injected intravenously with *Bb*ss organisms produced any specific antibodies, and only 30-KELA-unit background level was measured. In individual serum samples collected on the final day, antibody levels ranged from 13.7 to 59.8 (average 29.8 ± 20.2) KELA units (Fig. 4b).

**Fig. 5:**
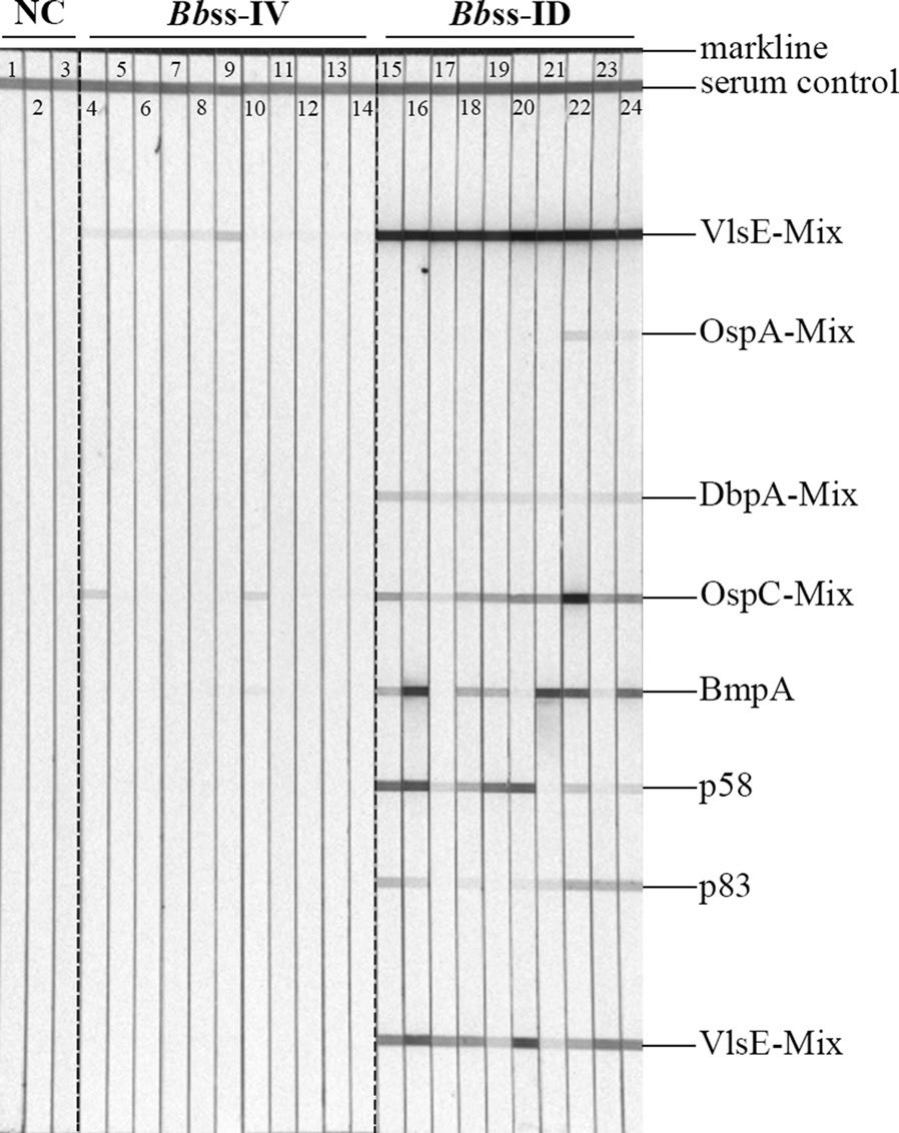
Representative IgG line immunoblots of individual serum samples from immunocompetent C3H/HeOuJ mice injected with host-adapted *Bbss*. Lanes show antibody signals of individual serum samples. Lanes 1–3: NC, plasma samples from three non-inoculated mice that served as a negative control group; Lanes 4–14: *Bb*ss-IV, plasma collected from mice that received *Bbssvia* strict intravenous injection; Lanes: 15–24: *Bbss*-ID, plasma collected from mice that received *Bbssvia* intradermal injection

As shown in Fig. 5, individual serum samples from eleven immunocompetent, ID-injected mice reacted on a line immunoblot (LIA) with the following antigens, which are indicative of infection with borreliae belonging to the *Bb* complex: a strong signal with VlsE mix (10/10); strong/moderate signals with OspC mix (8/10), BmpA (7/10) and p58 (5/10); and weak signals with DbpA mix (10/10) and p83 (8/10). On the contrary, none of the serum samples from the IV-injected mice produced specific signals during line immunoblotting.

## Discussion

*Borrelia burgdorferi* sensu stricto (*Bb*ss) is a tick-transmitted spirochete that causes Lyme borreliosis in humans and animals. In a murine model, the ability of *Bb*ss organisms to disseminate *via* the hematogenous route in a mammalian host was investigated. The fitness of *Bb*ss to survive in blood and utilize this body fluid as a vehicle was compared to that of *B. persica*, a representative of tick-transmitted spirochetes that cause relapsing fever in humans and animals and are known for their ability to thrive in the blood circulation. However, before tick-transmitted microorganisms enter the blood stream, they must adapt to the new environment after they have been deposited into the dermis of the new host. Unlike insects (e.g. mosquitoes), ticks, as arachnids are pool feeders and deliver their germ-loaded saliva into the surrounding tissue, from where bacteria need to access the microvasculature if they depend on the circulation for further survival and growth. Alternatively, disease-causing organisms may stay in the tissue and use cells and extracellular matrices as passageways to distant body sites. During the time after the tick bite that spirochetes spend in the host’s dermis, the microorganisms undergo substantial reshaping of their outer surface coating [47], which is why host-adapted spirochetes were utilized for the experiments presented in this paper. Furthermore, the smallest injuries during inoculation, which offer spirochete access to tissues, are a starting point for further tissue-based colonization. Therefore, we opted to use microsurgery to obtain controlled access through the large jugular vein to avoid any damage to the endothelium lining the vasculature during strict intravenous spirochete injection. In this way, we avoided endothelial damage and unintentional deposition of spirochete in host tissue that are likely to occur following injection into the mouse’s tail vein. This infection model allowed us to clarify whether *Bp* and *Bb*ss organisms are capable of entering and subsequently leaving the blood stream to colonize distant sites in mammals. The successful recultivation of *Borrelia* organisms from tissues sufficiently distant from the inoculation site is ample evidence of satisfactory virulence and dissemination capability of these spirochetes, especially in light of the discussion on disseminating and non-disseminating *B. burgdorferi* strains [22, 23, 48, 49]. The evaluation of all parameters, including tissue culture, PCR and antibody response during the experiments, indicated that the blood-bound *Bb*ss spirochetes do not leave the blood stream and that the circulatory system is an impasse for *Bb*ss, whereas *Bp* organisms are able to penetrate the vessels and disseminate further to distant tissues. For *Bp*, the brain tissue may serve as a reservoir [50].

As outlined above, borreliae must adapt physiologically to two different host environments during the course of its enzootic cycle between the arthropod vector and mammalian host [2, 47, 51]. Spirochete adaptation to the mammal milieu was necessary in our study to avoid the interference of any antigen (e.g. OspA) chiefly derived from *in vitro* cultivation [52]. Therefore, immunodeficient NOD-SCID mice were utilized to generate the host-adapted *Bp* and *Bb*ss spirochetes because these mice lack mature T and B cells and complement activity [32], which allow higher bacterial burdens in the blood stream than those affected by the immune responses of immunocompetent animals [24, 34, 53]. Support for the contention regarding antigen expression *in vivo* comes from our further experiment with immunocompetent mice, which were infected with these host-adapted *Bb*ss organisms but failed to produce specific antibodies against OspA (Fig. 5).

In the blood of immunodeficient NOD-SCID mice, *Bp* was detectable on day 1 after injection and at a higher concentration than *Bb*ss, which was detectable on day 2 after inoculation of culture-derived borreliae. Up to 8.8 ± 1.2 × 10^6^ spirochetes per ml blood (on day 17) was observed in the case of *Bp*; *Bb*ss concentrations, however, climaxed at 4.9 ± 3.3 × 10^4^ cells/ml on day 7 pi (Fig. 2). Hence, it seems that *Bp* is more robust than *Bb*ss in adaptation and replication in the blood of immunodeficient mice given that a 10-fold lower inoculum of *Bp* organisms than *Bb*ss (10^6^ organisms/mouse) was injected into these animals. On day 12 or day 10 pi, spirochetes were present in various tissues of infected NOD-SCID mice, as shown by *in vitro* cultivation and qPCR (Table 3), indicating robust spirochete proliferation in tissues of immunodeficient animals.

*Bp* spirochetes in the blood of immunocompetent C3H/HeOuJ mice were detectable up to 24 days after either ID or strict IV inoculation with 7.2 × 10^5^ host-adapted *Bp* organisms. Fluctuations in spirochete numbers during this period (Fig. 3) might be due to the appearance of specific antibodies, which increased until day 21, followed by a slight decline until day 24 (Fig. 4a). Spirochetemia for *Bp* was not documented beyond day 24, while at the same time, high antibody levels increased until day 49/50. However, these antibodies were not able to eliminate *Bp* infection in brain tissue (Table 4), which is an immune-privileged site where no tissue damage or clinical signs were induced by *Bp* [12]. Consistent with other TBRF *Borrelia* spp. (e.g. *B. duttonii*, *B. turicatae*, *B. crocidurae* and *B. hispanica*) that show residual brain infection [54], *Bp* spirochetes, though faced with mammalian host immunity, also show this tropism regardless of the inoculation route (ID or IV). Obviously, *Bp* can survive and multiply in the blood stream, and the microvasculature does not present a barrier for *Bp*.

Similarly, when *Bb*ss was inoculated intradermally, specific antibodies were produced during the infection (Figs. 4, 5), and viable spirochetes were recovered from various tissues samples (Table 5). In sharp contrast, neither *Bb*ss DNA nor motile spirochetes were detected in the blood samples. Inoculated host-adapted *Bb*ss spirochetes (3.5 × 10^3^ or 2.8 × 10^3^ per animal) initiated a robust infection and were able to invade distant tissue sites in immunocompetent mice following ID inoculation. Although single tissue samples showed culture-positive but PCR-negative results for *Bb*ss (Table 5), the possible reason might be that *Bb*ss-specific DNA in the samples was present at a very low level, which might have been below the detection limit of the qPCR applied in this study. This hypothesis is supported by the finding that even one single viable spirochete may be recovered by culture from a tissue sample, whereas it is not possible to detect the borrelial DNA in a single cell by PCR [55]. *Bb*ss appears to only infrequently target the brain tissue but prefers to persist in tissues such as the skin, bladder, joint, and heart of immunocompetent mice following ID challenge, consistent with prior reports dealing with LB infection in humans and animals [56, 57].

In the experiments presented here, *Bb*ss spirochetes were inoculated *via* a strict intravenous route at a dose of 2.8 to 3.5 × 10^3^ organisms per animal. This infection may mimic the spirochetemia phase, which is detected infrequently in blood samples from patients during early LB infection [22, 58]. Our results (Table 5) suggest that these *Bb*ss organisms are cleared by the animals’ innate immunity [59, 60]. The lack of *Bb*ss-specific antibodies in the course of the intravenous injection strongly suggests that these spirochetes did not even multiply or provide sufficient antigen to stimulate the host B cell population. However, LB spirochetes inoculated into the skin can persist and multiply locally and disseminate to distal sites [61]. During dissemination, the blood seems to play no role because *Bb*ss organisms were not detected in any blood sample. Instead, skin and connective soft tissues probably serve as critical intermediate media for spirochete spread. This hypothesis is further supported by the observations that LB borreliae were only occasionally or not at all re-isolated from peripheral blood of immunocompetent animals even when a persistent infection was established, regardless if it was initiated by a low-inoculum (10^3^, 10^4^, intradermal injection) or high-inoculum (10^8^, subcutaneous injection) dose [34, 53, 62]. In addition, Shih et al. [56] stated that the expanding EM seems to represent the advancing front of a wave of the spirochetal organisms ‘dermatogenously’ migrating outward from the deposition site in the skin of the human host. They and others [63] have also highlighted the issue that prompt excision or topical treatment with antibiotics applied in the site of murine skin shortly after tick exposure or needle inoculation can avert systemic infection. Moreover, immunosuppression with dermocorticoid clobetasol reactivated LB *Borrelia* abundance in the skin tissue, while the blood still remained spirochete negative [61]. In contrast, high levels of spirochetemia were achieved in the course of a TBRF infection (persistently infected brain tissues as source for spirochetes) when a state of immunosuppression was initiated [50]. Furthermore, the large numbers of genes (e.g. decorin-binding proteins (Dbps), such as DbpA and DbpB, and the fibronectin-binding protein BBK32), which are selectively expressed by LB spirochetes in mammalian hosts, need to be considered, which contribute to the spirochetes’ dissemination and colonization of target tissues [64].

Borreliae are frequently found in certain tissues since they depend on essential substrates, such as N-acetylglucosamine (NAG) [65]. In mammals, NAG is a substrate for hyaluronan synthesis [66] and consequently present in certain tissues at varying concentrations. Thus, it is likely that *Borrelia* organisms invade organs, e.g. joints and other tissues containing NAG. However, a subpopulation of *Borrelia* organisms remains in the skin and consequently is able to infect ticks attached to the skin.

Despite the results presented here, an earlier study with a similar experimental setup still came to the conclusion that *Bb*ss disseminates hematogenously. Gabitzsch et al. [67] supposedly intravenously injected the spirochete inocula. Uncontrolled injection, as outlined earlier, carries the risk of depositing spirochetes in the surrounding tissues when retracting the needle from the animal. Interestingly, the authors state clearly that in cases where intravenous injection was not successful, an IP injection was performed [67]. Obviously, their results were obtained by IP rather than by IV injection, thus may lead to a misinterpretation of the data and do not clarify how *Bb*ss disseminates from the injection site to the distant tissues. In our study, microsurgery avoided vessel damage and spillover of *Borrelia*-containing blood into the surrounding tissues (Additional file 1: Video S1). Other studies [68, 69] utilized huge doses of *Bb*ss spirochetes (culture-derived and up to 4 × 10^8^ per animal) for IV inoculation, arguing that hematogenous spread is the result of few interactions between spirochetes and endothelial cells under shear force conditions within a short time period (5–45 min). The puzzling results of these studies with enormous inocula were also contested by other authors [70], since the maximal concentration of LB spirochetes achievable in suitable culture medium is ~ 10^8^–10^9^ per ml [71, 72]. In comparison, only a small number of spirochetes per injection site (up to 10^3^ organisms) is deposited into the dermis of mice after tick attachment [61, 73–76]. Hence, not only the strict IV inoculation but also a reasonable inoculum dose plays the most crucial role in establishing a reliable murine model and drawing a realistic conclusion. Our relatively low inoculum size for *Bb*ss may faithfully reflect the actual pathophysiological conditions during natural infection after a tick bite. We also speculate that in the case of *Bp*, our infection dose of 7.2 × 10^5^ organisms per mouse approximates human and animal infections in the field since the observed spirochetemia in our experimental animals closely mimics the situation reported for blood samples retrieved from human TBRF cases and other laboratory experimental animals [6–9].

Another controversial aspect regarding the spirochetal dissemination route is the question of whether blood transfusion is safe in the context of *Bp* and *Bb*ss transmission. Relapsing fever spirochetes have been well documented in a few blood transfusion-mediated cases [77–79], as well as other tick-borne agents, such as *Ehrlichia* spp. [80] and *Babesia* spp. [81]. In addition, *B. hermsii*, a TBRF-causing species, has been shown to induce spirochetemia after intravenous inoculation into immunocompetent animals [82, 83]. *Babesia miyamotoi*, a newly recognized TBRF spirochete transmitted by *Ixodes* spp. ticks in the USA and Europe, where LB is endemic, have also been demonstrated to produce transfusion-transmitted infections in immunocompetent mice, suggesting the possibility of transfusion transmission of this species in people [84]. However, to date, there is no report of natural blood transfusion-mediated transmission of *B. burgdorferi*, despite the ability of spirochetes to survive in blood samples for prolonged time periods under blood bank conditions [85–87]. Our findings of the inability of *Bb*ss to leave the blood stream may answer this question, and we argue that the risk of acquiring LB by blood transfusion should be considered nonexistent. However, a transfusion of TBRF-causing *Bp* is highly problematic.

In future studies, it would be of interest if an experiment was performed with additional *Borrelia* species to evaluate the potential strain differences. Here, we report on a proof of principle. However, it is not expected that various strains of *Bb*ss should behave differently when they are injected directly into the blood stream, as performed in this study. Other researchers, (e.g. [88–90]), who have reported on the variable behavior of different *Bb*ss strains, did not apply a strict intravenous injection protocol, as applied in this study. Thus, the data obtained from this study cannot be compared with the results described in the publications mentioned above. The deposition of *Bb*ss into the skin and subsequent colonization of the injection site will subsequently establish a depot effect. *Borrelia* organisms will multiply in the days following the injection, and a large number of spirochetes have the chance to enter into the circulation for an extended time period, resulting in an accumulation of spirochetes in the blood until they are cleared by the host. In contrast, in a single spirochete bolus (100 µl volume) that is injected intravenously, the number of organisms will not increase over time (according to the hypothesis stated in this paper). Most likely, the number will decrease due to the host’s non-specific immune response. As a consequence, such a small number of spirochetes in a given blood sample is difficult to detect. A potential drawback of this study is the lack of an experiment that checks whether a strict IV protocol compromises the infectivity of *Bb*ss. In the view of the authors, this possibility is unlikely.

## Conclusions

In conclusion, our results provide new insights into the dissemination routes of *Bp* and *Bb*ss. *Bp* is able to translocate in mammalian hosts *via* the blood stream, whereas *Bb*ss is unable to spread hematogenously and establish an infection *via* that route, consequently relying on tissue migration instead. However, our studies on LB are limited to the *Bb*ss species. Further studies that include *B. afzelii* and *B. garinii* spirochetes, which cause different clinical manifestations of LB [91–93], are necessary to explore further dissemination pathways to better understand how *Borrelia* spp. interact with host cells.

## Supplementary information

**Additional file 1: Video S1.** Strict intravenous inoculation *via* the mouse jugular vein. The intravenous inoculation of pooled blood containing host-adapted *Borrelia* organisms into the mouse jugular vein was recorded using a stereomicroscope equipped with a Leica MC170 HD camera. The inoculation, from making the incision on the chin through suturing of the incision, of each mouse required approximately 60 min. The whole video in duration was edited for displaying important procedures.

## Data Availability

Data supporting the conclusions of this article are included within the article and its additional file. Further data of interest will be available from the corresponding author upon request.

## Abbreviations

LD: Lyme disease
*Bb*: *Borrelia burgdorferi*
*Bb*ss: *Borrelia burgdorferi* sensu stricto
IV: intravenous
TBRF: tick-borne relapsing fever
IP: intraabdominal

## References

[1] Felsenfeld O (1971). *Borrelia*: strains, vectors, human and animal borreliosis.

[2] Schwan TG, Piesman J (2002). Vector interactions and molecular adaptations of Lyme disease and relapsing fever spirochetes associated with transmission by ticks. Emerg Infect Dis.

[3] Rebaudet S, Parola P (2006). Epidemiology of relapsing fever borreliosis in Europe. FEMS Immunol Med Microbiol.

[4] Assous MV, Wilamowski A (2009). Relapsing fever borreliosis in Eurasia—forgotten, but certainly not gone!. Clin Microbiol Infect.

[5] Oshaghi MA, Rafinejad J, Choubdar N, Piazak N, Vatandoost H, Telmadarraiy Z (2011). Discrimination of relapsing fever *Borrelia persica* and *Borrelia microtti* by diagnostic species-specific primers and polymerase chain reaction-restriction fragment length polymorphism. Vector Borne Zoonotic Dis.

[6] Kutsuna S, Kawabata H, Kasahara K, Takano A, Mikasa K (2013). The first case of imported relapsing fever in Japan. Am J Trop Med Hyg.

[7] Colin de Verdière N, Hamane S, Assous MV, Sertour N, Ferquel E, Cornet M (2011). Tickborne relapsing fever caused by *Borrelia persica,* Uzbekistan and Tajikistan. Emerg Infect Dis.

[8] Baneth G, Nachum-Biala Y, Halperin T, Hershko Y, Kleinerman G, Anug Y (2016). *Borrelia persica* infection in dogs and cats: clinical manifestations, clinicopathological findings and genetic characterization. Parasites Vectors.

[9] Rafinejad J, Choubdar N, Oshaghi M, Piazak N, Satvat T, Mohtarami F (2011). Detection of *Borrelia persica* infection in *Ornithodoros tholozani* using PCR targeting *rrs* gene and xenodiagnosis. Iran J Public Health.

[10] Assous MV, Wilamowski A, Bercovier H, Marva E (2006). Molecular characterization of tickborne relapsing fever *Borrelia,* Israel. Emerg Inf Dis.

[11] Addamiano L, Babudieri B (1957). Research on spirochaetal strains isolated in Jordan. Bull World Health Org.

[12] Schwarzer S, Overzier E, Hermanns W, Baneth G, Straubinger RK (2016). *Borrelia persica* infection in immunocompetent mice—a new tool to study the infection kinetics *in vivo*. PLoS Negl Trop Dis.

[13] Greene CE, Straubiner R, Levy S, Greene CE (2012). Borreliosis. Infectious diseases of the dog and cat.

[14] Steere AC (1997). Diagnosis and treatment of Lyme arthritis. Med Clin N Am.

[15] Steere AC (1995). Musculoskeletal manifestations of Lyme disease. Am J Med.

[16] Borchers AT, Keen CL, Huntley AC, Gershwin ME (2015). Lyme disease: a rigorous review of diagnostic criteria and treatment. J Autoimmun.

[17] Rizzoli A, Hauffe HC, Carpi G, Vourc’h G, Neteler M, Rosa R (2011). Lyme borreliosis in Europe. Euro Surveillance.

[18] Steere AC, Schoen RT, Taylor E (1987). The clinical evolution of Lyme arthritis. Ann Intern Med.

[19] Steere AC (1989). Lyme disease. N Engl J Med.

[20] Steere AC, Strle F, Wormser GP, Hu LT, Branda JA, Hovius JW (2016). Lyme borreliosis. Nat Rev Dis Primers.

[21] Goodman JL, Bradley JF, Ross AE, Goellner P, Lagus A, Vitale B (1995). Bloodstream invasion in early Lyme disease: results from a prospective, controlled, blinded study using the polymerase chain reaction. Am J Med.

[22] Wormser GP, Liveris D, Nowakowski J, Nadelman RB, Cavaliere LF, McKenna D (1999). Association of specific subtypes of *Borrelia burgdorferi* with hematogenous dissemination in early Lyme disease. J Infect Dis.

[23] Ojaimi C, Mulay V, Liveris D, Iyer R, Schwartz I (2005). Comparative transcriptional profiling of *Borrelia burgdorferi* clinical isolates differing in capacities for hematogenous dissemination. Infect Immun.

[24] Ristow LC, Bonde M, Lin YP, Sato H, Curtis M, Wesley E (2015). Integrin binding by *Borrelia burgdorferi* P66 facilitates dissemination but is not required for infectivity. Cell Microbiol.

[25] Hyde JA (2017). *Borrelia burgdorferi* keeps moving and carries on: a review of borrelial dissemination and invasion. Front Immunol.

[26] Benach JL, Bosler EM, Hanrahan JP, Coleman JL, Habicht GS, Bast TF (1983). Spirochetes isolated from the blood of two patients with Lyme disease. N Engl J Med.

[27] Oksi J, Marttila H, Soini H, Aho H, Uksila J, Viljanen MK (2001). Early dissemination of *Borrelia burgdorferi* without generalized symptoms in patients with erythema migrans. APMIS.

[28] Ruzić-Sabljić E, Arnez M, Lotric-Furlan S, Maraspin V, Cimperman J, Strle F (2001). Genotypic and phenotypic characterisation of *Borrelia burgdorferi sensu lato* strains isolated from human blood. J Med Microbiol.

[29] Straubinger RK, Straubinger AF, Harter L, Jacobson RH, Chang YF, Summers BA (1997). *Borrelia burgdorferi* migrates into joint capsules and causes an up-regulation of interleukin-8 in synovial membranes of dogs experimentally infected with ticks. Infect Immun.

[30] Motameni AR, Bates TC, Juncadella IJ, Petty C, Hedrick MN, Anguita J (2005). Distinct bacterial dissemination and disease outcome in mice subcutaneously infected with *Borrelia burgdorferi* in the midline of the back and the footpad. FEMS Immunol Med Microbiol.

[31] Berglund J, Eitrem R, Ornstein K, Lindberg A, Ringnér Å, Elmrud H (1995). An epidemiologic study of Lyme disease in southern Sweden. N Engl J Med.

[32] Milsom CC, Lee CR, Hackl C, Man S, Kerbel RS (2013). Differential post-surgical metastasis and survival in SCID, NOD-SCID and NOD-SCID-IL-2Rgamma(null) mice with parental and subline variants of human breast cancer: implications for host defense mechanisms regulating metastasis. PLoS ONE.

[33] LaRocca T, Benach J. The important and diverse roles of antibodies in the host response to *Borrelia* infections. In: Curr Top Microbiol Immunol. Specialization and complementation of humoral immune responses to infection. Berlin: Springer; 2008. p. 63–103. 10.1007/978-3-540-73900-5_4 18080415

[34] Hodzic E, Feng S, Freet KJ, Barthold SW (2003). *Borrelia burgdorferi* population dynamics and prototype gene expression during infection of immunocompetent and immunodeficient mice. Infect Immun.

[35] Schaible U, Kramer M, Museteanu C, Zimmer G, Mossmann H, Simon M (1989). The severe combined immunodeficiency (scid) mouse. A laboratory model for the analysis of Lyme arthritis and carditis. J Exp Med.

[36] Xu Q (2006). Mice-general information. A handbook of mouse models of cardiovascular disease.

[37] Schneider K, Dhein S, Mohr FW, Delmar M (2005). Anaesthesia of laboratory animals. Practical methods in cardiovascular research.

[38] Barthold SW, Beck DS, Hansen GM, Terwilliger GA, Moody KD (1990). Lyme borreliosis in selected strains and ages of laboratory mice. J Infect Dis.

[39] Krupka I, Knauer J, Lorentzen L, OʼConnor TP, Saucier J, Straubinger RK (2009). *Borrelia burgdorferi sensu lato* species in Europe induce diverse immune responses against C6 peptides in infected mice. JCI Insight.

[40] Schwarzer S, Margos G, Overzier E, Fingerle V, Baneth G, Straubinger RK (2015). *Borrelia persica*: *in vitro* cultivation and characterization *via* conventional PCR and multilocus sequence analysis of two strains isolated from a cat and ticks from Israel. Ticks Tick Borne Dis.

[41] Pollack RJ, Telford SR, Spielman A (1993). Standardization of medium for culturing Lyme disease spirochetes. J Clin Microbiol.

[42] Straubinger RK, Straubinger AF, Summers BA, Erb HN, Harter L, Appel MJ (1998). *Borrelia burgdorferi* induces the production and release of proinflammatory cytokines in canine synovial explant cultures. Infect Immun.

[43] Straubinger RK (2000). PCR-based quantification of *Borrelia burgdorferi* organisms in canine tissues over a 500-day postinfection period. J Clin Microbiol.

[44] Barth C, Straubinger RK, Krupka I, Muller E, Sauter-Louis C, Hartmann K (2014). Comparison of different diagnostic assays for the detection of *Borrelia burgdorferi*-specific antibodies in dogs. Vet Clin Pathol.

[45] Appel MJ, Allan S, Jacobson RH, Lauderdale TL, Chang YF, Shin SJ (1993). Experimental Lyme disease in dogs produces arthritis and persistent infection. J Infect Dis.

[46] Shin SJ, Chang YF, Jacobson RH, Shaw E, Lauderdale TL, Appel MJ (1993). Cross-reactivity between *B. burgdorferi* and other spirochetes affects specificity of serotests for detection of antibodies to the Lyme disease agent in dogs. Vet Microbiol.

[47] Schwan TG, Piesman J, Golde WT, Dolan MC, Rosa PA (1995). Induction of an outer surface protein on *Borrelia burgdorferi* during tick feeding. Proc Natl Acad Sci USA.

[48] Lagal V, Postic D, Ruzic-Sabljic E, Baranton G (2003). Genetic diversity among *Borrelia* strains determined by single-strand conformation polymorphism analysis of the *ospC* gene and its association with invasiveness. J Clin Microbiol.

[49] Seinost G, Dykhuizen DE, Dattwyler RJ, Golde WT, Dunn JJ, Wang IN (1999). Four clones of *Borrelia burgdorferi sensu stricto* cause invasive infection in humans. Infect Immun.

[50] Larsson C, Andersson M, Pelkonen J, Guo BP, Nordstrand A, Bergström S (2006). Persistent brain infection and disease reactivation in relapsing fever borreliosis. Microbes Infect.

[51] De Silva AM, Fikrig E (1997). Arthropod- and host-specific gene expression by *Borrelia burgdorferi*. J Clin Invest.

[52] Cassatt DR, Patel NK, Ulbrandt ND, Hanson MS (1998). DbpA, but not OspA, is expressed by *Borrelia burgdorferi* during spirochetemia and is a target for protective antibodies. J Infect.

[53] Liu N, Montgomery RR, Barthold SW, Bockenstedt LK (2004). Myeloid differentiation antigen 88 deficiency impairs pathogen clearance but does not alter inflammation in *Borrelia burgdorferi*-infected mice. Infect Immun.

[54] Cadavid D, Barbour AG (1998). Neuroborreliosis during relapsing fever: review of the clinical manifestations, pathology, and treatment of infections in humans and experimental animals. Clin Infect Dis.

[55] Barbour AG (1984). Isolation and cultivation of Lyme disease spirochetes. Yale J Biol Med.

[56] Shih CM, Pollack RJ, Telford SR, Spielman A (1992). Delayed dissemination of Lyme disease spirochetes from the site of deposition in the skin of mice. J Infect Dis.

[57] Divan A, Casselli T, Narayanan SA, Mukherjee S, Zawieja DC, Watt JA (2018). *Borrelia burgdorferi* adhere to blood vessels in the dura mater and are associated with increased meningeal T cells during murine disseminated borreliosis. PLoS ONE.

[58] Wormser GP (2006). Hematogenous dissemination in early Lyme disease. Wien Klin Wochenschr.

[59] Kraiczy P, Skerka C, Kirschfink M, Zipfel PF, Brade V (2001). Mechanism of complement resistance of pathogenic *Borrelia burgdorferi* isolates. Int Immunopharmacol.

[60] de Taeye SW, Kreuk L, van Dam AP, Hovius JW, Schuijt TJ (2013). Complement evasion by *Borrelia burgdorferi*: it takes three to tango. Trends Parasitol.

[61] Grillon A, Westermann B, Cantero P, Jaulhac B, Voordouw MJ, Kapps D (2017). Identification of *Borrelia* protein candidates in mouse skin for potential diagnosis of disseminated Lyme borreliosis. Sci Rep.

[62] Simon M, Schaible U, Wallich R, Kramer M (1991). A mouse model for *Borrelia burgdorferi* infection: approach to a vaccine against Lyme disease. Immunol Today.

[63] Knauer J, Krupka I, Fueldner C, Lehmann J, Straubinger RK (2011). Evaluation of the preventive capacities of a topically applied azithromycin formulation against Lyme borreliosis in a murine model. J Antimicrob Chemother.

[64] Pal U, Fikrig E (2003). Adaptation of *Borrelia burgdorferi* in the vector and vertebrate host. Microbes Infect.

[65] Fraser CM, Casjens S, Huang WM, Sutton GG, Clayton R, Lathigra R (1997). Genomic sequence of a Lyme disease spirochaete, *Borrelia burgdorferi*. Nature.

[66] Połubinska A, Cwalinski J, Baum E, Bręborowicz A (2013). *N*-Acetylglucosamine modulates function of the skin fibroblasts. Int J Cosmet Sci.

[67] Gabitzsch ES, Piesman J, Dolan MC, Sykes CM, Zeidner NS (2006). Transfer of *Borrelia burgdorferi ss* infection *via* blood transfusion in a murine model. J Parasitol.

[68] Moriarty TJ, Norman MU, Colarusso P, Bankhead T, Kubes P, Chaconas G (2008). Real-time high resolution 3D imaging of the Lyme disease spirochete adhering to and escaping from the vasculature of a living host. PLoS Pathog.

[69] Norman MU, Moriarty TJ, Dresser AR, Millen B, Kubes P, Chaconas G (2008). Molecular mechanisms involved in vascular interactions of the Lyme disease pathogen in a living host. PLoS Pathog.

[70] Hyde JA, Weening EH, Chang M, Trzeciakowski JP, Hook M, Cirillo JD (2011). Bioluminescent imaging of *Borrelia burgdorferi in vivo* demonstrates that the fibronectin-binding protein BBK32 is required for optimal infectivity. Mol Microbiol.

[71] Aguero-Rosenfeld ME, Wang G, Schwartz I, Wormser GP (2005). Diagnosis of Lyme borreliosis. Clin Microbiol Rev.

[72] Pappas CJ, Iyer R, Petzke MM, Caimano MJ, Radolf JD, Schwartz I (2011). *Borrelia burgdorferi* requires glycerol for maximum fitness during the tick phase of the enzootic cycle. PLoS Pathog.

[73] Kern A, Collin E, Barthel C, Michel C, Jaulhac B, Boulanger N (2011). Tick saliva represses innate immunity and cutaneous inflammation in a murine model of Lyme disease. Vector Borne Zoonotic Dis.

[74] Burkot TR, Wirtz RA, Luft B, Piesman J (1993). An OspA antigen-capture enzyme-linked immunosorbent assay for detecting North American isolates of *Borrelia burgdorferi* in larval and nymphal *Ixodes dammini*. J Clin Microbiol.

[75] Golde WT, Kappel KJ, Dequesne G, Feron C, Plainchamp D, Capiau C (1994). Tick transmission of *Borrelia burgdorferi* to inbred strains of mice induces an antibody response to P39 but not to outer surface protein A. Infect Immun.

[76] De Silva AM, Fikrig E (1995). Growth and migration of *Borrelia burgdorferi* in Ixodes ticks during blood feeding. Am J Trop Med Hyg.

[77] Hira P, Husein S (1979). Some transfusion-induced parasitic infections in Zambia. J Hyg Epidemiol Microbiol Immunol.

[78] Wang C, Lee C (1936). Malaria and relapsing fever following blood transfusion including the report of a case of congenital transmission of relapsing fever. Chin Med J.

[79] Nadelman RB, Wormser GP, Sherer C (1990). Blood transfusion-associated relapsing fever. Transfusion.

[80] McQuiston JH, Childs JE, Chamberland ME, Tabor E (2000). Transmission of tick-borne agents of disease by blood transfusion: a review of known and potential risks in the United States. Transfusion.

[81] Mintz ED, Anderson JF, Cable RG, Hadler JL (1991). Transfusion-transmitted babesiosis: a case report from a new endemic area. Transfusion.

[82] Dickinson GS, Sun G, Bram RJ, Alugupalli KR (2014). Efficient B cell responses to *Borrelia hermsii* infection depend on BAFF and BAFFR but not TACI. Infect Immun.

[83] Benoit VM, Petrich A, Alugupalli KR, Marty-Roix R, Moter A, Leong JM (2010). Genetic control of the innate immune response to *Borrelia hermsii* influences the course of relapsing fever in inbred strains of mice. Infect Immun.

[84] Krause PJ, Hendrickson JE, Steeves TK, Fish D (2015). Blood transfusion transmission of the tick-borne relapsing fever spirochete *Borrelia miyamotoi* in mice. Transfusion.

[85] Badon SJ, Fister RD, Cable RG (1989). Survival of *Borrelia burgdorferi* in blood products. Transfusion.

[86] Johnson SE, Swaminathan B, Moore P, Broome CV, Parvin M (1990). *Borrelia burgdorferi*: survival in experimentally infected human blood processed for transfusion. J Infect Dis.

[87] Nadelman RB, Sherer C, Mack L, Pavia CS, Wormser GP (1990). Survival of *Borrelia burgdorferi* in human blood stored under blood banking conditions. Transfusion.

[88] Wang G, Ojaimi C, Wu H, Saksenberg V, Iyer R, Liveris D (2002). Disease severity in a murine model of Lyme borreliosis is associated with the genotype of the infecting *Borrelia burgdorferi sensu stricto* strain. J Infect Dis.

[89] Dolan MC, Piesman J, Schneider BS, Schriefer M, Brandt K, Zeidner NS (2004). Comparison of disseminated and nondisseminated strains of *Borrelia burgdorferi sensu stricto* in mice naturally infected by tick bite. Infect Immun.

[90] Wang G, Ojaimi C, Iyer R, Saksenberg V, McClain SA, Wormser GP (2001). Impact of genotypic variation of *Borrelia burgdorferi sensu stricto* on kinetics of dissemination and severity of disease in C3H/HeJ mice. Infect Immun.

[91] Stanek G, Wormser GP, Gray J, Strle F (2012). Lyme borreliosis. Lancet.

[92] Veinović G, Filipić B, Stanković J (2013). Isolation, cultivation, and *in vitro* susceptibility testing of *Borrelia burgdorferi sensu lato*: a review. Arch Biol Sci.

[93] Coipan EC, Jahfari S, Fonville M, Oei GA, Spanjaard L, Takumi K (2016). Imbalanced presence of *Borrelia burgdorferi sensu lato* multilocus sequence types in clinical manifestations of Lyme borreliosis. Infect Genet Evol.

